# Supplementary material for: Thermostable chaperone-based polypeptide biosynthesis: Enfuvirtide model product quality and protocol-related impurities
Source: PLoS One. 2023 Jun 8;18(6):e0286752. doi: 10.1371/journal.pone.0286752 (PMC10249821; doi:10.1371/journal.pone.0286752)
Supplement: S1 File — (DOCX) [file pone.0286752.s001.docx]

# Supporting information S1

GroEL-Enfuvirtide fusion protein sequence:

MAKILVFDEAARRALERGVNAVANAVKVTLGPRGRNVVLEKKFGSPTITKDGVTVAKEVELEDHLENIGAQLLKEVASKTNDVAGDGTTTATVLAQAIVREGLKNVAAGANPLALKRGIEKAVEAAVEKIKALAIPVEDRKAIEEVATISANDPEVGKLIADALEKVGKEGIITVEESKSLETELKFVEGYQFDKGYIGSMYTSLIHSLIEESQNQQEKNEQELLELDKWASLWNWFMEFVTNPETLEAVLEDAFILIVEKKVSNVRELLPILEQVAQTGKPLLIIAEDVEGEALATLVVNKLRGTLSVAAVKAPGFGDRRKELLKDIAAVTGGTVISEELGFKLENATLSLLGRAERVRITKDETTIVGGKGKKEDIEARINGIKKELETTDSEYAREKLQERLAKLAGGVAVIRVGAATETELKEKKHRFEDALNATRAAVEEGIVPGGGVTLLRAISAVEELIKKLEGDEATGAKIVRRALEEPARQIAENAGYEGSVIVQQILAETKNPRYGFNAATGEFVDLVEAGIVDPAKVTRSALQNAASIGALILTTEAVVAEKPEKKESTPASAGAGDLDF

Table. All detected HCPs in GroEL-Enfuvirtide

| Protein | MW [kDa] | pI | #Peptides | SC [%] | Score | RMS90 [ppm] |
| --- | --- | --- | --- | --- | --- | --- |
| Elongation factor G OS=Escherichia coli (strain K12 / MC4100 / BW2952) GN=fusA PE=3 SV=1 | 77.5 | 5.2 | 15 | 42 | 811.1 | 1.52 |
| 60 kDa chaperonin OS=Escherichia coli O139:H28 (strain E24377A / ETEC) GN=groL PE=3 SV=1 | 57.3 | 4.8 | 13 | 39.4 | 762.3 | 1.6 |
| Elongation factor Tu 1 OS=Escherichia coli O139:H28 (strain E24377A / ETEC) GN=tuf1 PE=3 SV=1 | 43.3 | 5.3 | 9 | 19.5 | 310.9 | 1.26 |
| Purine nucleoside phosphorylase DeoD-type OS=Escherichia coli (strain ATCC 8739 / DSM 1576 / Crooks) GN=deoD PE=3 SV=1 | 25.9 | 5.4 | 8 | 49 | 277.1 | 2 |
| Enolase OS=Escherichia coli (strain K12 / MC4100 / BW2952) GN=eno PE=3 SV=1 | 45.6 | 5.3 | 7 | 33.8 | 484.7 | 1.77 |
| 30S ribosomal protein S6 OS=Escherichia coli O139:H28 (strain E24377A / ETEC) GN=rpsF PE=3 SV=1 | 15.2 | 5.2 | 6 | 45 | 239.1 | 1.89 |
| Polyribonucleotide nucleotidyltransferase OS=Escherichia coli (strain K12 / MC4100 / BW2952) GN=pnp PE=3 SV=1 | 77.1 | 5.1 | 6 | 12.7 | 209.4 | 1.79 |
| Chaperone protein DnaK OS=Escherichia coli (strain ATCC 8739 / DSM 1576 / Crooks) GN=dnaK PE=3 SV=1 | 69.1 | 4.8 | 5 | 13.2 | 405.1 | 2.33 |
| 30S ribosomal protein S1 OS=Escherichia coli O157:H7 GN=rpsA PE=3 SV=1 | 61.1 | 4.9 | 5 | 11.1 | 245.4 | 2.7 |
| 50S ribosomal protein L7/L12 OS=Escherichia coli O139:H28 (strain E24377A / ETEC) GN=rplL PE=3 SV=1 | 12.3 | 4.6 | 5 | 19 | 245.3 | 2.15 |
| Elongation factor Ts OS=Escherichia coli (strain K12 / MC4100 / BW2952) GN=tsf PE=3 SV=1 | 30.4 | 5.2 | 5 | 23.7 | 193.4 | 2.78 |
| Trigger factor OS=Escherichia coli (strain UTI89 / UPEC) GN=tig PE=3 SV=1 | 47.8 | 4.8 | 4 | 16.6 | 313.7 | 2.24 |
| S-ribosylhomocysteine lyase OS=Escherichia coli O7:K1 (strain IAI39 / ExPEC) GN=luxS PE=3 SV=1 | 19.4 | 5.3 | 4 | 24 | 160.9 | 2.66 |
| Protein GrpE OS=Escherichia coli O9:H4 (strain HS) GN=grpE PE=3 SV=1 | 21.8 | 4.7 | 4 | 35.5 | 157.1 | 2.16 |
| DNA-binding protein H-NS OS=Escherichia coli (strain K12) GN=hns PE=1 SV=2 | 15.5 | 5.4 | 3 | 35.8 | 102.2 | 2.72 |
| Phosphoenolpyruvate-protein phosphotransferase OS=Escherichia coli (strain K12) GN=ptsI PE=1 SV=1 | 63.5 | 4.8 | 3 | 8.2 | 97.6 | 1.63 |
| Acetate kinase OS=Escherichia coli (strain K12) GN=ackA PE=1 SV=1 | 43.3 | 5.8 | 3 | 12.5 | 93.4 | 1.73 |
| Universal stress protein A OS=Escherichia coli (strain K12) GN=uspA PE=1 SV=2 | 16.1 | 5.1 | 3 | 34 | 92.1 | 1.13 |
| NADP-dependent malic enzyme OS=Escherichia coli (strain K12) GN=maeB PE=1 SV=1 | 82.4 | 5.3 | 3 | 5.7 | 42 | 1.66 |
| FKBP-type peptidyl-prolyl cis-trans isomerase SlyD OS=Escherichia coli (strain K12) GN=slyD PE=1 SV=1 | 20.8 | 4.9 | 2 | 19.9 | 152.4 | 1.64 |
| Fe/S biogenesis protein NfuA OS=Escherichia coli (strain K12 / MC4100 / BW2952) GN=nfuA PE=3 SV=1 | 21 | 4.5 | 2 | 15.2 | 121 | 1.28 |
| Dihydrolipoyllysine-residue acetyltransferase component of pyruvate dehydrogenase complex OS=Escherichia coli (strain K12) GN=aceF PE=1 SV=3 | 66.1 | 5.1 | 2 | 3.8 | 104.8 | 2.32 |
| Single-stranded DNA-binding protein OS=Escherichia coli (strain K12) GN=ssb PE=1 SV=2 | 19 | 5.4 | 2 | 12.9 | 103.6 | 3.29 |
| Alkyl hydroperoxide reductase subunit C OS=Escherichia coli (strain K12) GN=ahpC PE=1 SV=2 | 20.7 | 5 | 2 | 8 | 86.6 | 1.9 |
| Ribosome maturation factor RimM OS=Escherichia coli (strain K12 / MC4100 / BW2952) GN=rimM PE=3 SV=1 | 20.6 | 4.6 | 2 | 9.9 | 82.8 | 3.45 |
| Glutaredoxin 4 OS=Escherichia coli O157:H7 GN=grxD PE=3 SV=1 | 12.9 | 4.7 | 2 | 39.1 | 76.4 | 3.4 |
| ATP synthase subunit delta OS=Escherichia coli (strain K12 / MC4100 / BW2952) GN=atpH PE=3 SV=1 | 19.3 | 4.9 | 2 | 13 | 73.4 | 1.17 |
| S-adenosylmethionine decarboxylase proenzyme OS=Escherichia coli (strain K12 / MC4100 / BW2952) GN=speD PE=3 SV=1 | 30.4 | 5.2 | 2 | 9.8 | 69.1 | 2.49 |
| Aspartate-semialdehyde dehydrogenase OS=Escherichia coli (strain K12) GN=asd PE=1 SV=1 | 40 | 5.4 | 2 | 8.7 | 54.2 | 1.32 |
| Uracil phosphoribosyltransferase OS=Escherichia coli (strain K12 / MC4100 / BW2952) GN=upp PE=3 SV=1 | 22.5 | 5.3 | 2 | 14.9 | 47.1 | 0.15 |
| 33 kDa chaperonin OS=Escherichia coli (strain K12 / MC4100 / BW2952) GN=hslO PE=3 SV=1 | 32.5 | 4.4 | 2 | 10.3 | 45.6 | 2.3 |
| Nucleoid-associated protein YbaB OS=Escherichia coli (strain K12 / MC4100 / BW2952) GN=ybaB PE=3 SV=1 | 12 | 5 | 2 | 31.2 | 44.8 | 2.1 |
| Aspartate 1-decarboxylase OS=Escherichia coli (strain K12 / MC4100 / BW2952) GN=panD PE=3 SV=1 | 13.8 | 5.8 | 2 | 26.2 | 40.6 | 2.56 |
| Acyl carrier protein OS=Escherichia coli (strain K12 / MC4100 / BW2952) GN=acpP PE=3 SV=1 | 8.6 | 4 | 1 | 42.3 | 113.4 | 3.08 |
| Thioredoxin reductase OS=Escherichia coli (strain K12) GN=trxB PE=1 SV=2 | 34.6 | 5.3 | 1 | 5 | 88.8 | 2.08 |
| 50S ribosomal protein L10 OS=Escherichia coli O139:H28 (strain E24377A / ETEC) GN=rplJ PE=3 SV=1 | 17.7 | 9 | 1 | 22.4 | 77.5 | 0.07 |
| Elongation factor P OS=Escherichia coli (strain K12 / MC4100 / BW2952) GN=efp PE=3 SV=1 | 20.6 | 4.9 | 1 | 8 | 76 | 0.3 |
| Inorganic pyrophosphatase OS=Escherichia coli (strain K12) GN=ppa PE=1 SV=2 | 19.7 | 5 | 1 | 25.6 | 67.8 | 2.03 |
| Biotin carboxyl carrier protein of acetyl-CoA carboxylase OS=Escherichia coli (strain K12) GN=accB PE=1 SV=1 | 16.7 | 4.6 | 1 | 10.9 | 67.2 | 1.67 |
| Purine nucleoside phosphoramidase OS=Escherichia coli (strain K12) GN=hinT PE=1 SV=1 | 13.2 | 5.7 | 1 | 18.5 | 56.7 | 2.07 |
| Pyruvate kinase I OS=Escherichia coli O157:H7 GN=pykF PE=3 SV=1 | 50.7 | 5.8 | 1 | 4.5 | 51.2 | 2.48 |
| Shikimate kinase 1 OS=Escherichia coli (strain K12 / DH10B) GN=aroK PE=3 SV=1 | 19.5 | 5.3 | 1 | 9.8 | 51.2 | 2.06 |
| Probable transcriptional regulatory protein YebC OS=Escherichia coli (strain K12) GN=yebC PE=1 SV=1 | 26.4 | 4.7 | 1 | 3.7 | 51 | 0.5 |
| dTDP-4-amino-4,6-dideoxy-D-glucose acyltransferase OS=Escherichia coli GN=vioB PE=1 SV=1 | 20.7 | 5 | 1 | 5.7 | 49.5 | 1.54 |
| UTP--glucose-1-phosphate uridylyltransferase OS=Escherichia coli (strain K12) GN=galU PE=1 SV=2 | 32.9 | 5.1 | 1 | 4 | 40.8 | 0.53 |
| Glutathione reductase OS=Escherichia coli (strain K12) GN=gor PE=1 SV=1 | 48.7 | 5.6 | 1 | 2.2 | 38.1 | 4.46 |
| 6,7-dimethyl-8-ribityllumazine synthase OS=Escherichia coli (strain K12 / MC4100 / BW2952) GN=ribH PE=3 SV=1 | 16.1 | 5.2 | 1 | 5.8 | 37.7 | 0.28 |
| DNA-binding protein StpA OS=Escherichia coli (strain K12) GN=stpA PE=1 SV=1 | 15.3 | 7.9 | 1 | 9 | 36.9 | 0.43 |
| Inosine-5'-monophosphate dehydrogenase OS=Escherichia coli (strain K12) GN=guaB PE=1 SV=1 | 52 | 6 | 1 | 5.1 | 35.4 | 1.59 |
| Xanthine phosphoribosyltransferase OS=Escherichia coli O6:K15:H31 (strain 536 / UPEC) GN=gpt PE=3 SV=1 | 17 | 5.8 | 1 | 7.2 | 34.8 | 2.77 |
| 5'-methylthioadenosine/S-adenosylhomocysteine nucleosidase OS=Escherichia coli (strain K12 / MC4100 / BW2952) GN=mtnN PE=3 SV=1 | 24.3 | 5.1 | 1 | 4.7 | 34.7 | 0.27 |
| Probable phosphatase YcdX OS=Escherichia coli O139:H28 (strain E24377A / ETEC) GN=ycdX PE=3 SV=1 | 26.8 | 5.6 | 1 | 3.7 | 32.1 | 1.1 |
| 50S ribosomal protein L1 OS=Escherichia coli O139:H28 (strain E24377A / ETEC) GN=rplA PE=3 SV=1 | 24.7 | 9.6 | 1 | 6.8 | 31.2 | 2.14 |
| UPF0381 protein YfcZ OS=Escherichia coli (strain K12) GN=yfcZ PE=3 SV=1 | 10.3 | 4.3 | 1 | 8.5 | 30.3 | 1.59 |
| 50S ribosomal protein L13 OS=Escherichia coli O139:H28 (strain E24377A / ETEC) GN=rplM PE=3 SV=1 | 16 | 9.9 | 1 | 7 | 28.7 | 0.12 |
| Ribosome maturation factor RimP OS=Escherichia coli (strain K12 / DH10B) GN=rimP PE=3 SV=1 | 16.6 | 4.6 | 1 | 6.7 | 26.6 | 1.69 |
| Bacterial non-heme ferritin OS=Escherichia coli (strain K12) GN=ftnA PE=1 SV=1 | 19.4 | 4.8 | 1 | 4.8 | 25 | 3.07 |
| PTS system glucose-specific EIIA component OS=Escherichia coli (strain K12) GN=crr PE=1 SV=2 | 18.2 | 4.7 | 1 | 14.8 | 24.1 | 0.36 |
| Outer membrane protein A OS=Escherichia coli (strain K12) GN=ompA PE=1 SV=1 | 37.2 | 6 | 1 | 2.3 | 23.2 | 2.86 |
| Glutathione synthetase OS=Escherichia coli (strain K12) GN=gshB PE=1 SV=1 | 35.5 | 5.1 | 1 | 3.2 | 22.6 | 1.96 |
| Glycerophosphodiester phosphodiesterase, periplasmic OS=Escherichia coli (strain K12) GN=glpQ PE=1 SV=2 | 40.8 | 5.4 | 1 | 4.5 | 22.4 | 1.1 |
| 50S ribosomal protein L16 OS=Escherichia coli O139:H28 (strain E24377A / ETEC) GN=rplP PE=3 SV=1 | 15.3 | 11.2 | 1 | 16.9 | 21.7 | 1.14 |
| Bifunctional protein GlmU OS=Escherichia coli O81 (strain ED1a) GN=glmU PE=3 SV=1 | 49.1 | 6 | 1 | 2.9 | 20.7 | 1.74 |
| Transcriptional regulatory protein BasR OS=Escherichia coli (strain K12) GN=basR PE=1 SV=3 | 25 | 5.7 | 1 | 8.6 | 19.7 | 1.91 |
| Ribulose-phosphate 3-epimerase OS=Escherichia coli (strain K12) GN=rpe PE=1 SV=1 | 24.5 | 5.1 | 1 | 4 | 19.6 | 4.36 |
| Phosphoheptose isomerase OS=Escherichia coli O7:K1 (strain IAI39 / ExPEC) GN=gmhA PE=3 SV=1 | 20.8 | 6 | 1 | 6.8 | 19.1 | 3.28 |
| Ornithine carbamoyltransferase OS=Escherichia coli O9:H4 (strain HS) GN=argI PE=3 SV=1 | 36.8 | 5.5 | 1 | 3 | 18.5 | 2.11 |
| Uridylate kinase OS=Escherichia coli O9:H4 (strain HS) GN=pyrH PE=3 SV=1 | 26 | 6.8 | 1 | 8.3 | 17.1 | 0.55 |
| 3-oxoacyl-[acyl-carrier-protein] synthase 2 OS=Escherichia coli (strain K12) GN=fabF PE=1 SV=2 | 43 | 5.7 | 1 | 1.9 | 16 | 2.95 |

DichroWeb-calculated approximations of secondary structures for sample at 20 and 65 C° and for standard at 20 C°

deltaE_smpl_before_melting.csv

| Helix1 | Helix2 | Strand1 | Strand2 | Turns | Unordered | Total |
| --- | --- | --- | --- | --- | --- | --- |
| 0.07 | 0.11 | 0.16 | 0.11 | 0.24 | 0.32 | 1.01 |

deltaE_smpl65.csv

| Helix1 | Helix2 | Strand1 | Strand2 | Turns | Unordered | Total |
| --- | --- | --- | --- | --- | --- | --- |
| 0.05 | 0.09 | 0.17 | 0.11 | 0.25 | 0.33 | 1 |

deltaE_std.tsv

| Helix1 | Helix2 | Strand1 | Strand2 | Turns | Unordered | Total |
| --- | --- | --- | --- | --- | --- | --- |
| 0.04 | 0.08 | 0.18 | 0.11 | 0.24 | 0.33 | 0.98 |

Second derivative NIR spectra


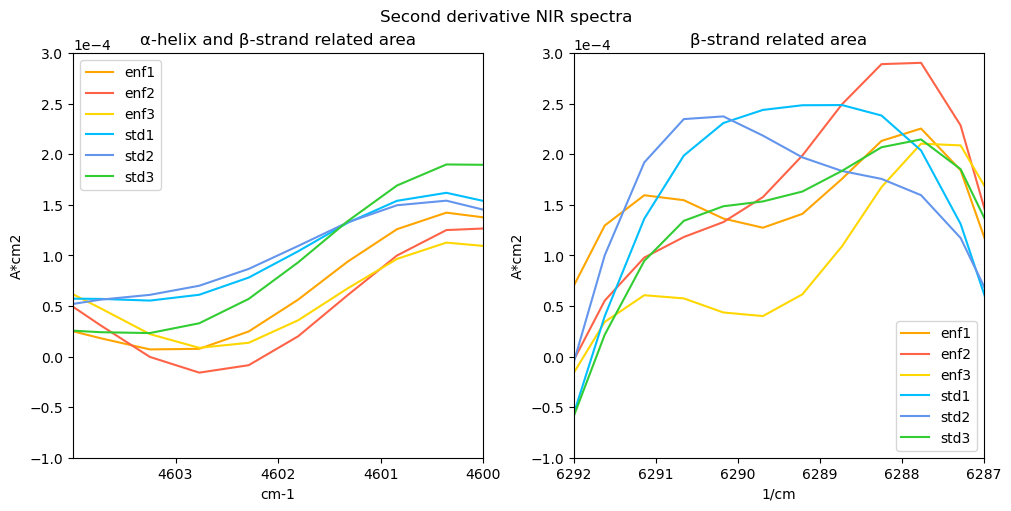


The samples (3 of each, standard and sample) were prepared at 25 mg/mL concentration in a 300 mM sodium phosphate buffer. Spectra were collected by FT/IR-6700typeA Spectrometer and ATR PRO ONE accessory (JASCO , Japan) by placing a drop of sample on diamond prism. Resulted spectra were smoothed and second derivative was calculated by scipy.signal.savgol_filter function of Scipy 1.10.1 package with window = XX and polyorder = X.

NIR spectra since the 80s is known as the source of secondary structure information for peptides and proteins[1]. Yet, the complexity of resulted data and high sample concentrations (up to 200 mg/mL in some studies)[2] has led to CD predominance as spectroscopic method for secondary structure estimation. We used NIR spectra as complementary method to confirm α-strand content difference for biosynthesised and chemically synthesised enfuvirtide. While several regions are known as secondary-structure related[3,4], we used 2 most valid points: 6289 cm–1 for β-sheet structure and 4602cm–1 for β-sheet and α-helical content simultaneously[5].

Resulting data potentially shows structural differences, yet β-strand related area shows excessive variability and no solid conclusions can be made.

1. Miyazawa M, Sonoyama M. Second Derivative near Infrared Studies on the Structural Characterisation of Proteins. J Infrared Spectrosc. 1998;6: A253–A257. doi:10.1255/jnirs.204

2. Izutsu K, Fujimaki Y, Kuwabara A, Hiyama Y, Yomota C, Aoyagi N. Near-infrared analysis of protein secondary structure in aqueous solutions and freeze-dried solids. J Pharm Sci. 2006;95: 781–789. doi:10.1002/jps.20580

3. Ishigaki M, Ozaki Y. Near-infrared spectroscopy and imaging in protein research. Vibrational Spectroscopy in Protein Research. Elsevier; 2020. pp. 143–176. doi:10.1016/B978-0-12-818610-7.00006-2

4. Beć KB, Grabska J, Huck CW. Near-Infrared Spectroscopy in Bio-Applications. Molecules. 2020;25: 2948. doi:10.3390/molecules25122948

5. Hsu LN, Lin TP, Sane SU. Near Infrared Spectroscopic Characterisation of Secondary Structure Content of Proteins. J Infrared Spectrosc. 2007;16: 437–444.
