# Supplementary material for: Thermostable chaperone-based polypeptide biosynthesis: Enfuvirtide model product quality and protocol-related impurities
Source: PLoS One. 2023 Jun 8;18(6):e0286752. doi: 10.1371/journal.pone.0286752 (PMC10249821; doi:10.1371/journal.pone.0286752)
Supplement: S2 File — (ZIP) [file pone.0286752.s002.zip › HCPs.docx]

| Substance name | «enf-b» |
| --- | --- |
| Date of analysis | 14.09.2022 |
| Quantity | 0.25 mg |
| Department that took the sample | OK |

**Used equipment**

| Name of equipment | №_identification_ | preparation instruction no. |
| --- | --- | --- |
| Tablet photometer "Infinite 200PRO" | 40704 | SOP-KO-3000-003-03 |
| Electronic scales "METLLER AE–240" | F/47144 | SOP-KO-3000-001-05 |
| Timer | ---- | SOP-KO-3000-035-05 |

The equipment used in this procedure has been prepared in accordance with their preparation procedures __________________

signature

**Reagents used**

| Substance name | The code | lot |
| --- | --- | --- |
| Reagent kit "E.coli Host Cell Proteins",  Cygnus Technologies, SHA | F410 | 180821A-1 |

**Sample preparation**

| Sample, mg | Buffer volume  (rN 7.5), ml | Solution concentration,  g/ml | Time (ultrasonic bath),  i |
| --- | --- | --- | --- |
| 0,25 | 0,25 | 1 | 5 |

**Calibration Graph Options**

| № | S_stand_,  ng/ml | Optical density, r.u. (450 nm) | | Type of equation | R^ |
| --- | --- | --- | --- | --- | --- |
|  |  | meaning | average |  |  |
| 1 | 0 | 0,0899  0,0876 | 0,0887 | 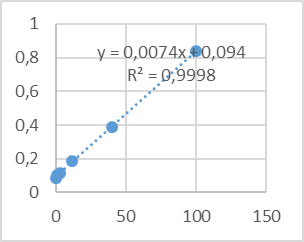 | 0,9996 |
| 2 | 1 | 0,1032  0,1041 | 0,1037 |  |  |
| 3 | 3 | 0,1161  0,1121 | 0,1141 |  |  |
| 4 | 12 | 0,1725  0,2066 | 0,1896 |  |  |
| 5 | 40 | 0,3465  0,4250 | 0,3910 |  |  |
| 6 | 100 | 0,8380  0,8372 | 0,8376 |  |  |

**Test results**

| A sample | Optical  density,  450nm, o.e. |  |  | Residual protein content  E.colli | |
| --- | --- | --- | --- | --- | --- |
|  |  | Average | S_oh_% | in sample | in the sample |
|  |  |  |  | ng/ml | ng/mg |
| **Enf-b** | 0,1332  0,1367  0,1386  0,1328 | 0,1353±0,005 | 2,1 | 5,58 | 5,58 |
| **Enf-b+ supplement St.100**  **(1:4)** | 0,3200  0,3015 | 0,3108±0,17 | 4,2 | 29,29  (Set manufacturer's requirement 15÷30) | |

According to the results of determining the content of immunoreactive polypeptides***E.coli*** in the "enf-b" pattern is ***5.58 ng/mg*** **(5,58 ppm).**

The kit manufacturer's requirements for “discovery” are met at the used dilution, which indicates the absence of an inhibition effect.
