# Supplementary material for: Thermostable chaperone-based polypeptide biosynthesis: Enfuvirtide model product quality and protocol-related impurities
Source: PLoS One. 2023 Jun 8;18(6):e0286752. doi: 10.1371/journal.pone.0286752 (PMC10249821; doi:10.1371/journal.pone.0286752)

**Cells**

The human T-lymphoblastoid cell line MT-4 (HTLV-1 transformed) was cultured in RPMI 1640 complete medium (Gibco, USA) supplemented with 10% fetal bovine serum (FBS; Sigma, USA), 1% GlutaMAX (Gibco, USA), and 1% antibiotics penicillin-streptomycin (Gibco, USA). Cells were passaged three times a week and cultured at a density of <0.5×106 cells/mL.

**Viruses**

The viral stock of the laboratory strain HIV-1 IIIB (NIH AIDS Reagent Program) was obtained during acute infection of MT-4 cells. The virus was stored in aliquots at -80°C.

**Antiviral assay**

The antiviral activity of the compounds against the HIV-1 IIIB strain in MT-4 cells was evaluated using a tetrazolium-based colorimetric assay. Briefly, this method is based on HIV-induced CPE in MT-4 cells 5 days post infection. The antiviral effects of the test compounds were directly correlated with the inhibition of virus-induced CPE by measuring cell viability using the MTT assay. MT-4 cells (6 x 105 cells/ml) were infected with the IIIB virus strain at 100 CCID50 in the presence of various compound dilutions. Protection against HIV-induced CPE was assessed using the MTT assay 5 days post-challenge.

**Results**

Table. The percentage of inhibition of HIV infection depending on the concentration of the drug. (Background-subtracted and normalized OD550)

| **Concentration (µМ)** | **Std** | | |  | **X** | | |
| --- | --- | --- | --- | --- | --- | --- | --- |
| ***10*** | 100 | 100 | 100 |  | 100 | 100 | 100 |
| ***1*** | 91,77312 | 75,01277 | 68,16556 |  | 81,349 | 72,35565 | 100 |
| ***0,1*** | 68,77874 | 65,09964 | 74,50179 |  | 70,2095 | 80,83802 | 69,80072 |
| ***0,01*** | 57,23045 | 54,77772 | 55,90189 |  | 15,63618 | 13,38784 | 21,56362 |
| ***0,001*** | 4,598876 | 3,270312 | 10,42412 |  | 0 | 11,2417 | 0 |
| ***0,0001*** | 19,62187 | 16,35156 | 16,65815 |  | 7,256004 | 8,482371 | 0 |
| ***0,00001*** | 2,452734 | 2,350537 | 0,919775 |  | 1,532959 | 0 | 4,496679 |


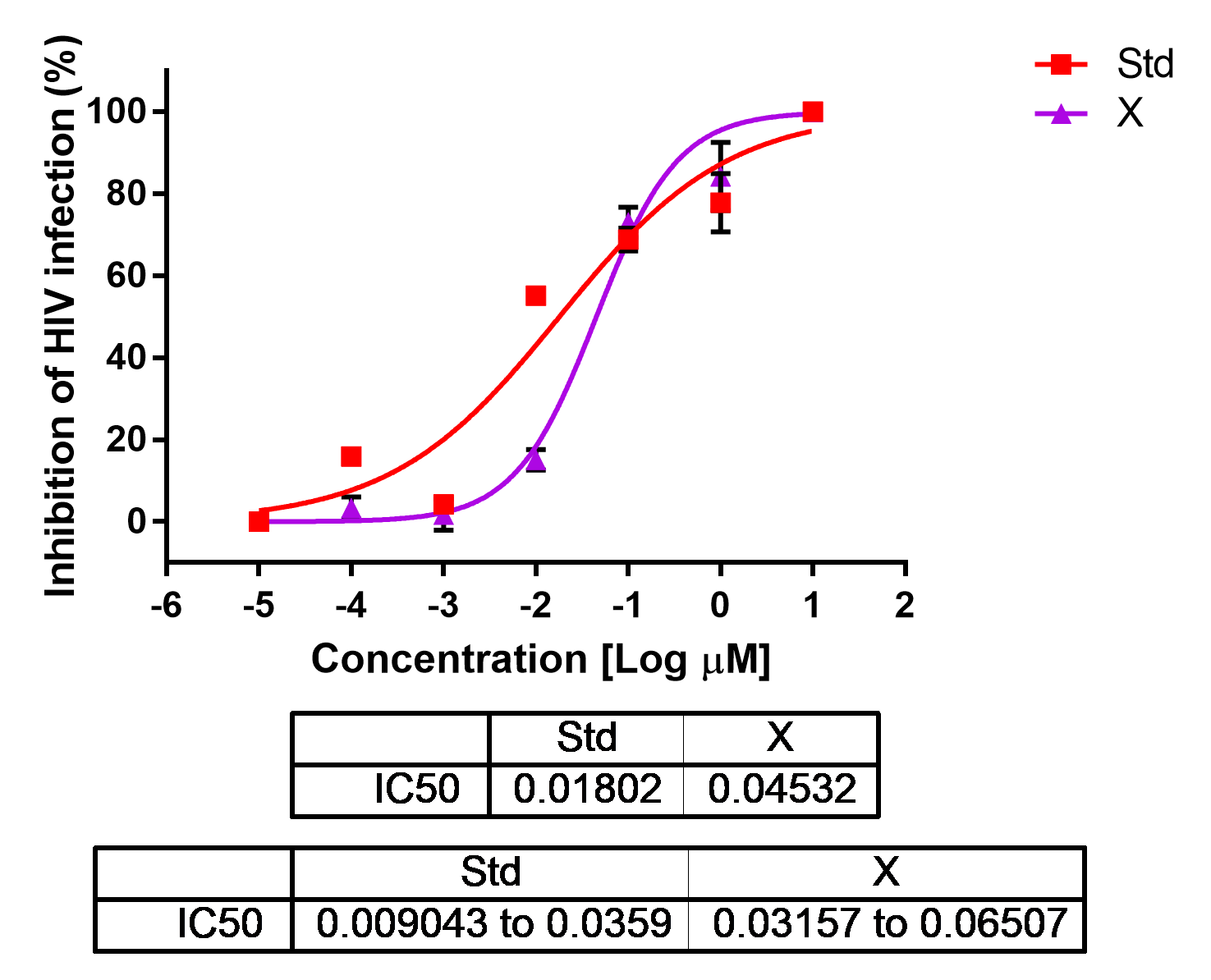

Supplement: S2 File — (ZIP) [file pone.0286752.s002.zip › IC50.docx]
