# Supplementary figures and images for: Thermostable chaperone-based polypeptide biosynthesis: Enfuvirtide model product quality and protocol-related impurities
Source: PLoS One. 2023 Jun 8;18(6):e0286752. doi: 10.1371/journal.pone.0286752 (PMC10249821; doi:10.1371/journal.pone.0286752)

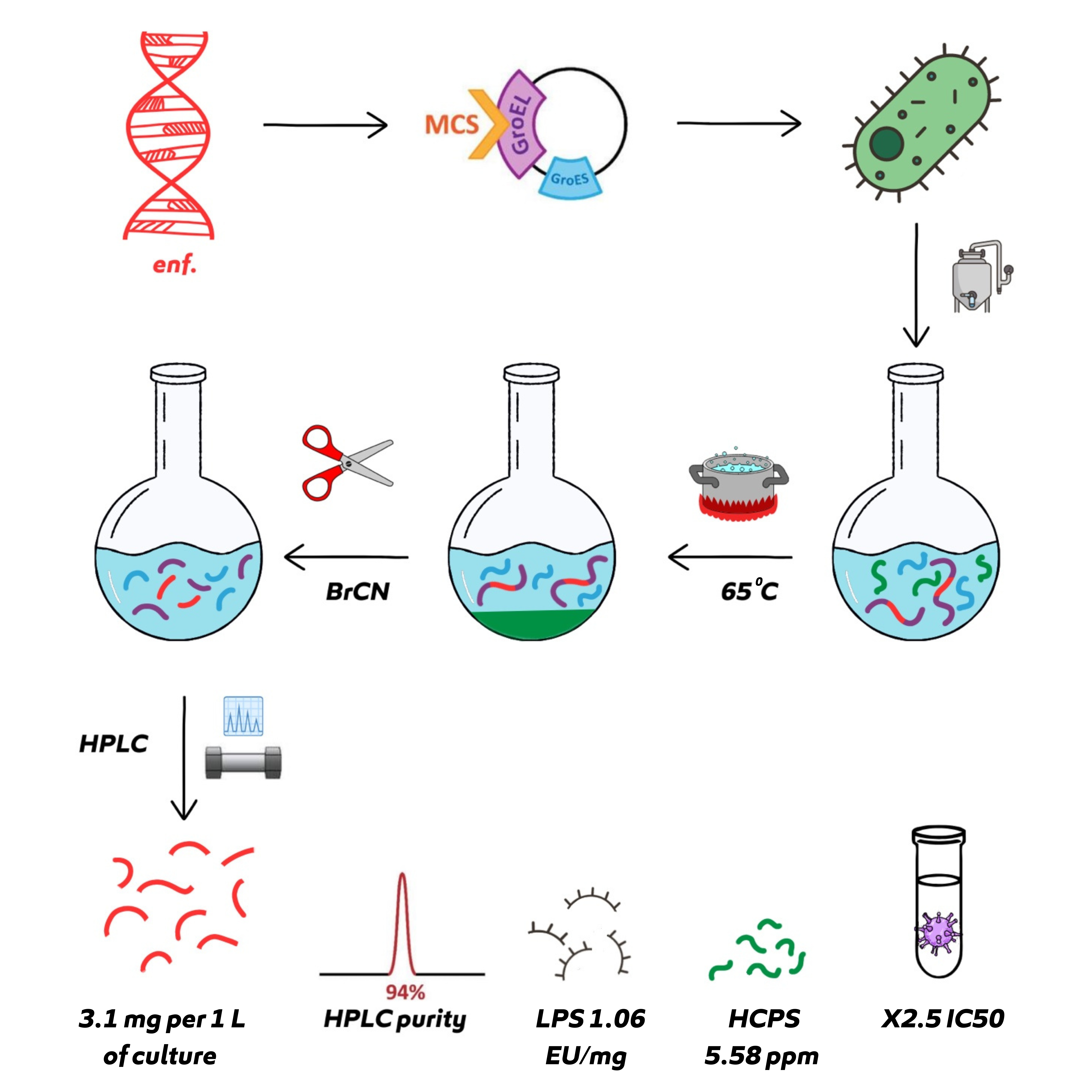

Supplement: S1 Graphical abstract — (TIFF) [file pone.0286752.s003.tiff]
